# Dr. John G. Hoeckh

**Published:** 1918-12

**Authors:** 


**Dr. John G. Hoeckh,** Buffalo 1907, died at his home in Buffalo, Oct. 5, of pneumonia, aged 33.

